# A Unique Egg Cortical Granule Localization Motif Is Required for Ovastacin Sequestration to Prevent Premature ZP2 Cleavage and Ensure Female Fertility in Mice

**DOI:** 10.1371/journal.pgen.1006580

**Published:** 2017-01-23

**Authors:** Bo Xiong, Yangu Zhao, Stephanie Beall, Anna Burkart Sadusky, Jurrien Dean

**Affiliations:** Laboratory of Cellular and Developmental Biology, NIDDK, National Institutes of Health, Bethesda, Maryland, United States of America; Cornell University, UNITED STATES

## Abstract

Monospermic fertilization is mediated by the extracellular zona pellucida composed of ZP1, ZP2 and ZP3. Sperm bind to the N-terminus of ZP2 which is cleaved after fertilization by ovastacin (encoded by *Astl*) exocytosed from egg cortical granules to prevent sperm binding. *Astl*^*Null*^ mice lack the post-fertilization block to sperm binding and the ability to rescue this phenotype with *Astl*^*mCherry*^ transgenic mice confirms the role of ovastacin in providing a definitive block to polyspermy. During oogenesis, endogenous ovastacin traffics through the endomembrane system prior to storage in peripherally located cortical granules. Deletion mutants of ovastacin^mCherry^ expressed in growing oocytes define a unique 7 amino acid motif near its N-terminus that is necessary and sufficient for cortical granule localization. Deletion of the 7 amino acids by CRISPR/Cas9 at the endogenous locus (*Astl*^*Δ*^) prevents cortical granule localization of ovastacin. The misdirected enzyme is present within the endomembrane system and ZP2 is prematurely cleaved. Sperm bind poorly to the zona pellucida of *Astl*^*Δ/Δ*^ mice with partially cleaved ZP2 and female mice are sub-fertile.

## Author Summary

Monospermic fertilization is essential for the onset of development. Egg cortical granules exocytose their contents after fertilization to prevent polyspermy by modifying the extracellular zona pellucida (ZP1, ZP2, ZP3). Little is known about the biology of these subcellular organelles which are unique to oocytes. Ovastacin, a zinc metalloendoprotease that cleaves ZP2 to prevent sperm binding, is a pioneer marker of mammalian cortical granules. ZP2 remains uncleaved in transgenic mice lacking ovastacin and sperm bind to the zona matrix independent of fertilization and cortical granule exocytosis. After documenting the rescue of the null phenotype with transgenic mice expressing fluorescently-tagged ovastacin, we defined a unique, well conserved, cortical granule localization motif using cRNA deletion mutants microinjected into mouse oocytes. The importance of the motif for localization to cortical granules was confirmed *in vivo* by deleting DNA encoding 7 amino acids of the endogenous locus with CRISPR/Cas9. Unexpectedly, mutant female mice were sub-fertile due to partial cleavage of ZP2 in the zona pellucida which prevented sperm from binding to ovulated eggs *in vitro* and *in vivo*. These observations offer unique insight into the molecular basis for translocation of proteins to cortical granules which is needed for successful, monospermic fertilization.

## Introduction

In mammals, gamete recognition initiates fertilization and the onset of development. Although an overwhelming number of sperm are deposited in the lower female reproductive tract at coitus, a limited number progress to encounter the one, or relatively few, ovulated eggs. For fertilization, sperm must bind and penetrate the extracellular zona pellucida to fuse with eggs in the ampulla of the oviduct. Equally important are mechanisms that limit fertilization to a single sperm to avoid polyspermy which is embryonic lethal. Following fertilization, there is an immediate block to gamete fusion at the egg plasma membrane [1, 2] that is independent of cortical granule exocytosis [3, 4]. Subsequent cortical granule exocytosis [5] blocks sperm binding to the zona pellucida [6], but the underlying molecular mechanisms remain obscure and we have little understanding of these subcellular organelles in mice.

Mammalian cortical granules, first described in hamster [7], are detected in mice starting in unilaminar follicles where immature oocytes are surrounded by a single layer of cuboidal granulosa cells. During oocyte-growth, cortical granules continuously arise from the Golgi apparatus [8] and require microfilaments to migrate to the periphery [9]. Concomitant with oocyte growth, the number of cortical granules increases to 6,000–8,000 and are uniformly distributed in the subcortex of full-grown, 80 μm oocytes [10]. However, cortical granule exocytosis is not yet enabled in these germinal vesicle (GV, nucleus) intact oocytes [11]. During meiosis I, a substantial cortical granule free domain appears in mice (25–40% of surface area), the first polar body is extruded and cortical granules decrease to ~4,000 [12]. Rather than early exocytosis, this cortical granule free domain reflects a redistribution of granules that is triggered by the peripheral migration of chromatin during meiosis I and formation of an actin cap [13]. Cortical granules become fully competent for exocytosis after completion of the first meiotic division in MII eggs [11, 14].

The egg is activated at fertilization and cortical granule exocytosis is triggered by release of Ca^+2^ from proximate, peripherally located stores in the endoplasmic reticulum [15]. Although precise details remain under investigation, cortical granule fusion with the egg plasma membrane involves SNARE-protein mediated pathways [16, 17]. Unlike regulated secretory granules in somatic cells (e.g., synaptic vesicles of neurons, zymogen granules of pancreatic acinar cells), cortical granules in oocytes are not renewed after exocytosis. Cortical granules contain a discrete set of proteins, albeit varying by report [18–20], including trypsin-like proteases [21–23], ovoperoxidase [24], N-acetylglycosaminidase [25], a 75 kD protein of unknown function [26] and, most recently, an astacin-like metalloendoprotease encoded by *Astl* [27]. The only documented function ascribed to cortical granules is the post-fertilization zona block to polyspermy that prevents sperm binding and penetration through the zona pellucida [22, 28].

The zona pellucida, composed of three (mouse) or four (human) glycoproteins (ZP1-4), surrounds growing oocytes, ovulated eggs and pre-implantation embryos [29, 30]. The N-terminus of ZP2^51-149^ has been defined as the zona ligand for sperm binding based on gain- and loss-of-function assays in transgenic mice [31, 32]. Ovastacin (435 amino acids) is an oocyte-specific member of the astacin-like family of Zn^+2^ metalloendoproteases that is synthesized as a zymogen with a signal peptide (1–23 aa) to direct it into the endomembrane system for ultimate storage in cortical granules. For enzymatic activity, astacins require removal of an N-terminal prosegment (24–85 aa) that runs in opposite direction to the future protein substrate [33]. The enzymatic active site of ovastacin is formed by glutamate and a single zinc atom complexed to three adjacent histidine residues (underlined, ^182^HELMHVLGFWH^192^). Following fertilization, ovastacin is exocytosed from egg cortical granules and cleaves ZP2 at ^166^LA^↓^DE^169^ after which sperm no longer bind. Ablation of *Astl* that encodes ovastacin, or mutation of the ZP2 cleavage site (*Zp2*^*Mut*^), prevents post-fertilization cleavage of ZP2 and sperm bind to the surface of the zona pellucida of 2-cell embryos despite fertilization and cortical granule exocytosis [27, 34].

Using mouse transgenesis, we rescue the *Astl*^*Null*^ phenotype by ectopic expression of *Astl*^*mCherry*^ and define a 7 amino acid motif required for ovastacin trafficking to cortical granules. Deletion of the cortical granule localization signal at the endogenous locus with CRISPR/Cas9 unexpectedly leads to premature modification of the egg’s zona pellucida and female sub-fertility.

## Results and Discussion

### *Astl*^*mCherry*^ transgenic mice provide an authentic biological marker for cortical granules

The zona pellucida and ovastacin proteins transverse the endomembrane system during oogenesis during which passage ZP2 remains intact [27]. Secreted ZP2 is incorporated into the extracellular zona pellucida surrounding ovulated eggs and is cleaved in the zona matrix surrounding 2-cell embryos [28]. In addition to a rapid, cortical-granule independent, post-fertilization block to gamete fusion [1, 2], ZP2 cleavage provides a definitive block to polyspermy in that sperm that do not bind cannot penetrate the zona matrix nor fuse with the egg plasma membrane [34].

**Fig 1:**
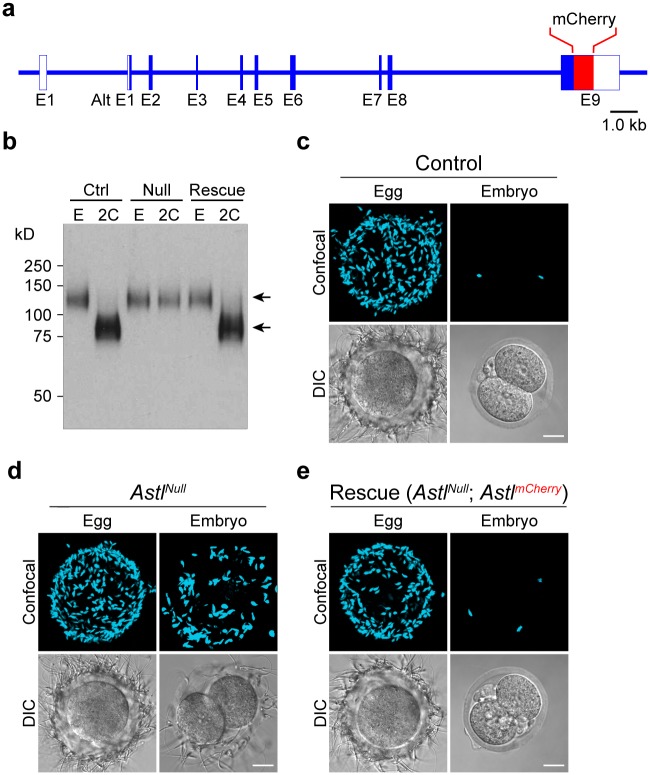
*Astl*^*mCherry*^ rescues the *Astl*^*Null*^ phenotype. **(a)** Schematic of the *Astl*^*mCherry*^ transgene. **(b)** Immunoblot of eggs (E) and 2-cell embryos (2C) from wild-type (Ctrl), *Astl*^*Null*^ (Null) and *Astl*^*Null*^; *Astl*^*mCherry*^ (Rescue) mice (10–15 eggs or embryos per lane). Primary antibody (M2c.2) binds to the C-terminus of ZP2 and detected intact (upper arrow) and cleaved (lower arrow) protein. Molecular mass (kD) on left. **(c-e)** Mouse sperm binding to eggs and two-cell embryos from: **(c)**, wild-type; **(d)**, *Astl*^*Null*^; and **(e)**, *Astl*^*mCherry*^ rescue mice. Confocal projections (upper) and DIC (lower) images were obtained after fixation and staining with Hoechst. Scale bar, 20 μm.

The single copy *Astl* gene encodes ovastacin (435 aa), an oocyte-specific metalloendoprotease [35] that has been located by immunohistochemistry in cortical granules at the periphery of growing oocytes and ovulated eggs. In *Astl*^*Null*^ mice that lack ovastacin, ZP2 remains intact in the zona surrounding 2-cell embryos [27]. To confirm that these observations reflect the absence of ovastacin, we established *Astl*^*mCherry*^ transgenic mice expressing ovastacin tagged with fluorescent mCherry at the C-terminus (Fig 1a, S1a and S1b Fig) and documented ovary-specific expression by RT-PCR (S1c Fig). The biological authenticity of the *Astl*^*mCherry*^ transgene *in vivo* was investigated by crossing *Astl*^*mCherry*^ with *Astl*^*Null*^ mice to generate a ‘rescue’ mouse line. The null phenotype was reversed in mice expressing the *Astl*^*mCherry*^ transgene in the *Astl*^*Null*^ background and ZP2 was cleaved in the zona pellucida surrounding 2-cell embryos (Fig 1b).

Sperm bind to the zona pellucida if ZP2 is uncleaved, independent of fertilization and cortical granule exocytosis [27, 34]. Thus, sperm did not bind to the zona matrix surrounding wild-type embryos (Fig 1c), but did bind to *Astl*^*Null*^ derived embryos (Fig 1d) in which ZP2 remains intact. However, sperm did not bind to 2-cell embryos derived from ‘rescue’ female mice (Fig 1e) in which ZP2 was cleaved (Fig 1b). The ability of *Astl*^*mCherry*^ transgene to rescue the *Astl*^*Null*^ phenotype is consistent with ovastacin acting as the cortical granule protease responsible for the post-fertilization cleavage of ZP2 that prevents sperm binding and provides a block to polyspermy. The *Astl*^*mCherry*^ mice also offer a useful platform for live-imaging that should provide additional insights into cortical granule biology.

### Localization of cortical granules before and after fertilization

**Fig 2:**
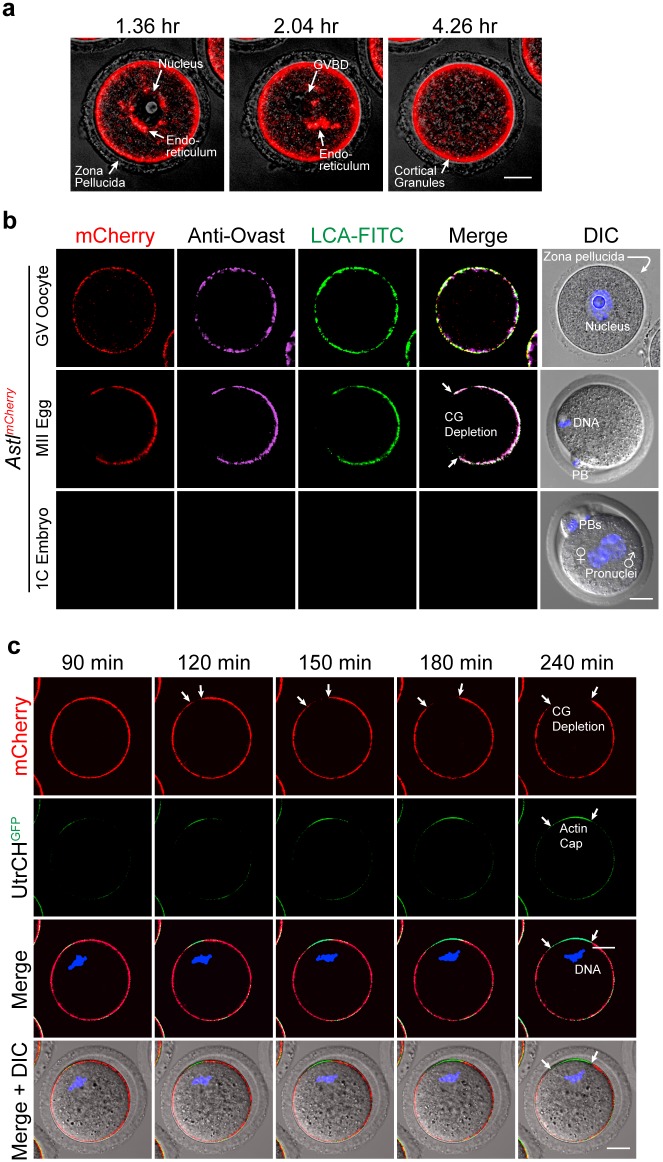
Localization of *Astl*^*mCherry*^ to cortical granules in transgenic mice. **(a)** Oocytes (70–75 μm) were isolated from *Astl*^*mCherry*^ transgenic mice and imaged in an environmental chamber (37°C, 5% CO_2_) by confocal microscopy during germinal vesicle (nucleus) breakdown (GVBD). **(b)** GV-intact oocytes (upper), ovulated metaphase II (MII) eggs (middle) and one cell (1C) embryos (lower) from *Astl*^*mCherry*^ transgenic mice were permeabilized, stained with antibody to ovastacin and with LCA-FITC to localize cortical granules and imaged with confocal and DIC microscopy. CG, cortical granule; PB, polar body. Scale bar, 20 μm. **(c)** GV-intact oocytes from *Astl*^*mCherry*^ transgenic mice were injected with cRNA encoding UtrCH^GFP^ (calponin homology domain of utrophin) to localize the actin cap associated with formation of the cortical granule free region during meiotic maturation. Live-images were obtained over time (4 hr) using confocal and DIC microscopy. Scale bars, 20 μm.

Prior to germinal vesicle (nucleus) breakdown (GVBD), cortical granules are uniformly present in the subcortex of fully grown oocytes [27]. To validate the intracellular trafficking of ovastacin to the cortex *in vivo*, intraovarian oocytes (up to 70 μm diameter) were isolated from *Astl*^*mCherry*^ transgenic mice and placed in an environmental chamber where confocal microscopy was used to obtain live images during GVBD. Most of the fluorescent signal was detected in peripherally located cortical granules. However, by increasing the sensitivity, ovastacin^mCherry^ was detected throughout the first 2 hr of culture in the peri-nuclear region where the endoplasmic reticulum (ER) is located (Fig 2a, left panel). Subsequently, during the early phases of GVBD, ovastacin^mCherry^ was present in close proximity to the dissolving nuclear membrane (Fig 2a, middle panel), but by 4 hr it was primarily located in the subcortex in the periphery of oocytes (Fig 2a, right panel). These observations were complemented in fixed samples in which ovastacin^mCherry^ partially co-localized with markers for the endoplasmic reticulum (GP73) and the Golgi apparatus (calregulin) in 70–75 μm growing oocytes (S2a Fig) which confirmed its presence in the endomembrane system. The absence of co-localization with the endosome pathway marker (EEA1) indicates little diversion into degradation pathways. Thus, we conclude that ovastacin follows the normal progression from the endoplasmic reticulum to the Golgi apparatus and is then sequestered in peripheral cortical granules.

Ovastacin^mCherry^, not present in wild-type GV-intact oocytes, co-localized with anti-ovastacin antibody staining and LCA consistent with the localization of endogenous ovastacin and ovastacin^mCherry^ in peripheral cortical granules (Fig 2b). During meiotic maturation, chromosomes move to the cortex at metaphase I where a cortical granule free domain (CGFD) develops (Fig 2b). The biological significance of CGFD is not understood, although it has been reported that sperm do not fuse with the egg plasma membrane in this region to protect the maternal genetic material [36]. However, whether cortical granules in this region are released or redistributed has not been fully resolved, although the latter has become the favored model [13].

To confirm that the presence of an actin cap correlated with the absence of cortical granules from the CGFD in the transgenic mice, cRNA encoding UtrCH-GFP, a green fluorescent protein tagged actin binding protein, was injected into oocytes from *Astl*^*mCherry*^ mice. Live imaging for 4 hr during meiotic maturation documented that the appearance of the actin cap and CGFD formation temporally coincided with complementary formation of the GFP (actin) and mCherry (ovastacin within cortical granules) signals (Fig 2c). To determine if formation of the CGFD was reversible, ovulated eggs from *Astl*^*mCherry*^ mice were incubated with CK-666 to stabilize the inactive form of the arp2/3 complex and depolymerize the actin cytoskeleton [37]. Within 3 hr, the cortical granules became uniformly distributed in the *Astl*^*mCherry*^ eggs and the metaphase plate migrates back towards the center of the cell (S2b Fig). Thus, we conclude that formation of the actin cap excludes cortical granules and results in a cortical granule free domain which has been reported to be physiologically induced by chromatin signaling [13].

Following fertilization and cortical granule exocytosis, ovastacin^mCherry^ was no longer detected in one-cell zygotes in fixed samples (Fig 2b). To detail this process, cortical granules were live-imaged with confocal microscopy during the 5 hr following fertilization. Sperm fused to eggs and cortical granules released ovastacin^mCherry^ 90–120 min after insemination. This process was complete by 150 min by which time the egg had extruded a second polar body (2^nd^ PB) to complete meiosis II. The sperm nucleus then decondensed (180 min) leading to formation of male and female pronuclei within 5 hr of insemination which was indicative of successful fertilization (S2c Fig). These observations are consistent with ovastacin being directed into the endomembrane system via its signal peptide and being transported to cortical granules in the periphery of growing oocytes. Ovastacin is rapidly released from cortical granules following fertilization and formation of the 1 cell zygote. However, the molecular basis for localization of ovastacin in cortical granules was unclear.

### Identification of a cortical granule localization motif

**Fig 3:**
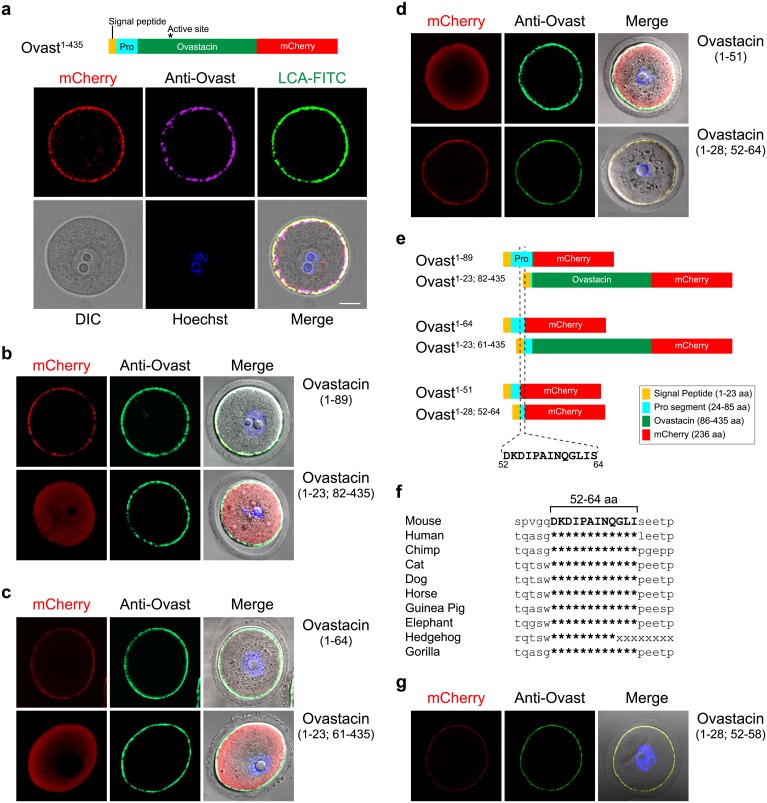
Ovastacin cortical granule localization motif. **(a)** Growing oocytes (60–65 μm) were injected with cRNA encoding an ovastacin^mCherry^ fusion protein (top) with a signal peptide (1–23 aa), a pro segment (24–85 aa) and the mature ovastacin enzyme (86–435 aa) containing an active site (asterisk, ^182^**HE**LM**H**VLGFW**H**^192^) fused to mCherry (236 aa). Six hr after injection, oocytes were fixed, permeabilized and stained with antibodies to ovastacin, LCA-FITC and Hoechst and imaged by confocal and DIC microscopy (bottom). Scale bar, 20 μm. **(b)** Same as **(a)** after injection of cRNA encoding either ovastacin 1–89 aa or ovastacin 1–23; 82–435 aa indicated in **(e)**. **(c)** Same as **(a)** after injection of cRNA encoding either ovastacin 1–64 aa or ovastacin 1–23; 61–435 aa indicated in **(e)**. **(d)** Same as **(a)** after injection of cRNA encoding either ovastacin 1–51 aa or ovastacin 1–28; 52–64 aa indicated in **(e)**. **(e)** Schematic representation of three pairs of complementary deletion constructs of ovastacin^mCherry^ fusion proteins injected into growing oocytes. Dotted lines indicate the minimal sequence (ovastacin^Δ52–64^) for localization of the fusion protein to cortical granules. **(f)** Primary amino acid sequences of ovastacin from 10 mammals were aligned to mouse ovastacin^47-69^. The conservation of mouse ovastacin^Δ52–64^ cortical granule localization signal is indicated by asterisks. **(g)** Same as **(a)** after injection of cRNA encoding ovastacin 1–28; 52–58.

To search a motif for cortical granule localization, cRNA encoding full-length ovastacin (1–435 aa) fused at the C-terminus with mCherry was injected into the cytoplasm of growing oocytes and transiently expressed for 6 hr as a positive control (Fig 3a). Subsequently, cRNA encoding deleted portions of ovastacin that retained the signal peptide, were injected into oocytes. Constructs encoding 1–89 aa, but not 82–435 aa (Fig 3b), localized to cortical granules as did constructs encoding 1–64 aa, but not 61–435 aa (Fig 3c). This initial delineation of a 41 aa motif (23–64 aa) was further refined by the observation that constructs with 52–64 aa, but not 1–51 aa, were sufficient for cortical granule localization (Fig 3d). Thus, ^52^DKDIPAINQGLIS^64^ is necessary and sufficient for the *in vitro* localization of a reporter protein to cortical granules (Fig 3e). Although well conserved among mammalian ovastacins (Fig 3f), the 13 amino acid motif was not identified in other proteins after a BLAST search of mouse databases.

### Deletion mutation of the cortical granule localizing motif of endogenous *Astl*

**Fig 4:**
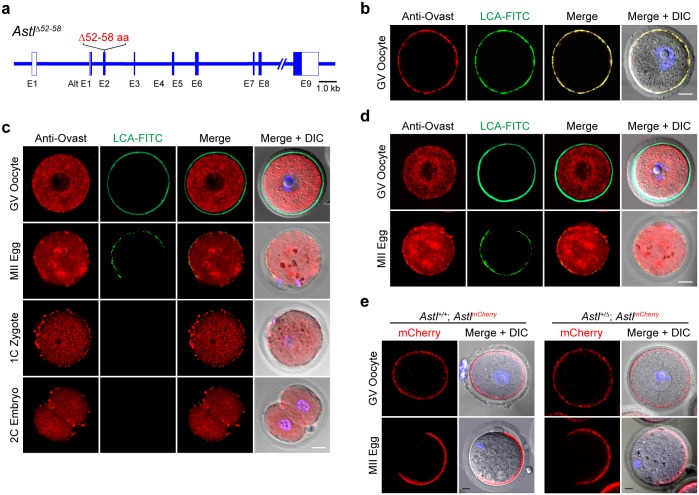
Ovastacin is not present in cortical granules after partial deletion of the localization motif. **(a)** Schematic representation of the CRISPR/Cas9 deletion mutation in *Astl* exon 2 that encodes ovastacin^Δ52–58^. **(b)** Wild-type GV-intact oocytes were fixed, permeabilized and stained with antibodies to ovastacin, LCA-FITC and imaged by confocal and DIC microscopy. **(c)** Same as **(b)** with GV-intact oocytes, ovulated MII eggs, 1-cell zygotes and 2-cell embryos from *Astl*^*Δ/Δ*^ mice. **(d)** Same as **(b)** with GV-intact oocytes and ovulated MII eggs from *Astl*^*+/Δ*^ mice. **(e)** GV-intact oocytes and ovulated MII eggs from *Astl*^*+/+*^; *Astl*^*mCherry*^ and *Astl*^*+/Δ*^; *Astl* ^*mCherry*^ mice were fixed, permeabilized and imaged by confocal and DIC microscopy. Scale bars, 20 μm.

The 13 aa cortical granule localizing motif is encoded by exons 2 and 3 of the endogenous *Astl* gene. To avoid disruption of RNA splicing, we elected to delete the first 7 aa (^52^DKDIPAI^58^) encoded entirely by exon 2 with a protospacer adjacent motif (PAM) sequence targeted by CRISPR/Cas9 (Fig 4a). To confirm the validity of this approach, cRNA encoding the signal peptide and ovastacin^52-58^ fused to mCherry were injected into oocytes. The localization of mCherry confirmed the sufficiency of the 7 aa to direct proteins to peripheral cortical granules (Fig 3g). To delete DNA encoding ^52^DKDIPAI^58^ at the endogenous *Astl* locus, Cas9 cRNA, sgRNA and HDR (homology directed repair) oligonucleotides (S3a and S3b Fig) were injected into zygotes, cultured to blastocysts, and transferred to uteri of pseudopregnant foster mothers. Of 7 pups screened by PCR (S3c Fig), two had bi-allelic mutations that were confirmed by DNA sequence (S3d and S3e Fig). In each line, the CRISPR/Cas9 mutation had been modified by homology directed repair to produce the desired deletion on one of the two endogenous alleles. Each line was crossed to wild-type mice to remove the non-desired mutant allele (either a single base pair insertion or deletion) and bred to homozygosity. Each expressed *Astl*^*Δ*^ alleles that encoded ovastacin lacking 52–58 aa (Fig 4a, ovastacin^Δ52–58^).

Using a monospecific antibody to the C-terminal region of the metalloendoprotease [27], ovastacin (accumulated during oocyte growth) was detected in cortical granules defined by staining with the LCA lectin in the periphery of wild-type GV oocytes (Fig 4b). In contrast, ovastacin was detected as punctate loci throughout the endomembrane system of oocytes and eggs (upper two rows of panels) from *Astl*^*Δ/Δ*^ female mice (Fig 4c) which could reflect either an inability to correctly traffic to cortical granules for storage or retrograde transport after reaching cortical granules. These results confirmed the *in vitro* transient assays in growing oocytes in which expression of mutant mCherry-tagged proteins was below the detection level of the antibody (Fig 3) and defined the 7 aa motif (ovastacin^Δ52–58^) as important for the correct intracellular trafficking of ovastacin. Following fertilization, cortical granule exocytosis was documented by the loss of LCA staining, but ovastacin remained diffusely present throughout the endomembrane system of zygotes and 2-cell embryos (Fig 4c, lower two rows of panels).

In oocytes and eggs from *Astl*^*+/Δ*^ mice, ovastacin was present both in the endomembrane system and at the periphery, where it partially co-localized with LCA in cortical granules (Fig 4d). To determine if the *Astl*^*Δ*^ allele was co-dominant, the *Astl*^*+/Δ*^ mice were crossed with *Astl*^*mCherry*^ transgenic mice to produce *Astl*^*+/Δ*^; *Astl*^*mCherry*^ transgenic mice. In these mice, ovastacin^mCherry^ lacks the deletion mutation and provides a proxy for the wild-type allele. Ovastacin^mCherry^ was detected in cortical granules located in the subcortex of oocytes and eggs (Fig 4e). Taken together, these observations define the ovastacin^Δ52–58^ protein as co-dominant with the wild-type protein and the absence of the cortical granule localization motif results in persistence of the mutant protein in the endomembrane system.

### Premature cleavage of ZP2 in *Astl*^*Δ/Δ*^ mice decreases female fertility

**Fig 5:**
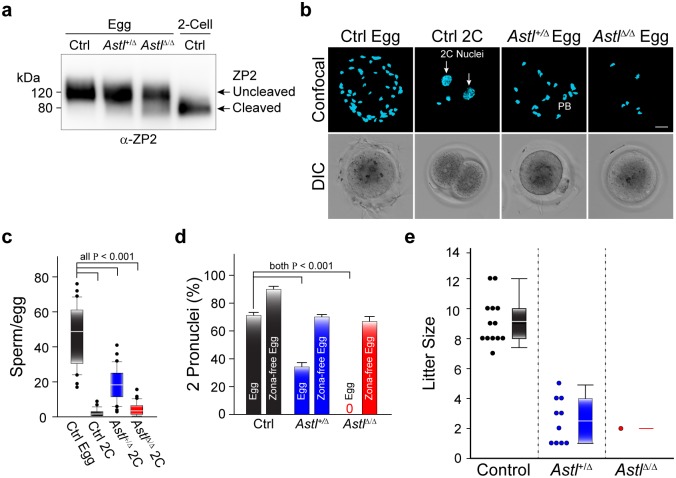
Premature cleavage of ZP2 affects fertility of *Astl* mutant mice. **(a)** Immunoblot of zonae pellucidae from wild-type (Ctrl), *Astl*^*+/Δ*^ and *Astl*^*Δ/Δ*^ ovulated eggs as well as wild-type 2C embryos (Ctrl) stained with a monoclonal antibody (M2c.2) that binds to the C-terminus of ZP2 and detected intact (upper arrow) and cleaved (lower arrow) protein. Molecular mass (kD) on left. **(b)** Sperm binding to wild-type eggs, 2C embryos, *Astl*^*+/Δ*^ and *Astl*^*Δ/Δ*^ eggs. Confocal projections (upper) and DIC (lower) images were obtained after fixation and staining nuclei with Hoechst. Arrows, nuclei; PB, polar body. Scale bar, 20 μm. **(c)** Average (± s.e.m) number of sperm bound to wild-type eggs (Ctrl), 2C embryos (Ctrl), *Astl*^*+/Δ*^ and *Astl*^*Δ/Δ*^ eggs imaged in **(b)**. N = 30 in each case. **(d)** *In vitro* fertilization with wild-type (left), *Astl*^*+/Δ*^ (center) and *Astl*^*Δ/Δ*^ (right) eggs with and without zonae pellucidae. Fertilization was determined by the presence of 2 pronuclei 12 hr after insemination. **(e)** Dot density (left) and associated box plots (right) of litters from wild-type (Control), *Astl*^*+/Δ*^ and *Astl*^*Δ/Δ*^ females co-caged with fertile male mice. The box includes the mean (horizontal line) and data between the 25^th^ and 75^th^ percentile. Error bars indicate the 90^th^ and 10^th^ percentiles and outliers are indicated by dots. Statistical differences in **c** and **d** were determined by 2-tailed Student’s T-test, P <0.001.

Zonae pellucidae isolated from eggs ovulated by wild-type, *Astl*^*+/Δ*^ and *Astl*^*Δ/Δ*^ female mice as well as from 2-cell embryos after mating wild-type mice were analyzed by immunoblot. As expected, ZP2 was uncleaved in wild-type eggs and completely cleaved in wild-type 2-cell embryos (Fig 5a). The partial cleavage of ZP2 observed in *Astl*^*+/Δ*^ eggs became more pronounced in *Astl*^*Δ/Δ*^ eggs and is similar to the ‘hardening’ reaction observed during *in vitro* fertilization in the absence of serum proteins [38].

Normally, sperm bind to the zona matrix surrounding eggs, but not 2-cell embryos. Compared to wild-type eggs, many fewer sperm bound *in vitro* to *Astl*^*+/Δ*^ eggs (18.9 ± 1.7 vs. 47.2 ± 3.0) and virtually no sperm (3.9 ± 0.7) bound to the zona pellucida surrounding *Astl*^*Δ/Δ*^ eggs (Fig 5b and 5c). *In vivo* fertilization of wild-type, *Astl*^*+/Δ*^ and *Astl*^*Δ/Δ*^ mice reflected these *in vitro* observations. After natural mating, eggs/embryos were recovered from the oviduct of female mice: 87.7 ± 2.3% of wild-type, 22.5 ± 4.0% of *Astl*^*+/Δ*^, but only 0.8 ± 0.8% of *Astl*^*Δ/Δ*^ eggs were fertilized (S1 Table).

To confirm that the block to fertilization was based on partially cleaved ZP2, fertility was assessed in the presence and absence of zonae pellucidae. After *in vitro* insemination with capacitated sperm, zona-intact *Astl*^*+/Δ*^ eggs had ~50% reduction in fertility compared to wild-type eggs and no *Astl*^*Δ/Δ*^ eggs were fertilized in these experiments. However, after removal of the zona pellucida matrix, the fertilization rates of zona-free *Astl*^*+/Δ*^ and *Astl*^*Δ/Δ*^ eggs were comparable (Fig 5d). Thus, we concluded that the partially cleaved ZP2 in the zona pellucida adversely affected fertilization.

The homozygous *Astl*^*Δ*^ allele had a profound effect on the fecundity of female mice. *In vivo* fertility of ovastacin^Δ52–58^ was assessed by co-caging wild-type, *Astl*^*+/Δ*^ and *Astl*^*Δ/Δ*^ females with male mice proven to be fertile. The number of litters from *Astl*^*+/Δ*^ was decreased compared to wild-type female mice (9 vs. 13) and the number of pups/litter produced by *Astl*^*+/Δ*^ was significantly lower than wild-type female mice (2.5 ± 0.8 vs. 9.2 ± 2.5). A single litter of 2 pups was obtained from eight *Astl*^*Δ/Δ*^ female mice (Fig 5e and S1 Table). Taken together, we conclude that ovastacin^Δ52–58^ does not properly traffic to cortical granules leading to premature cleavage of ZP2 which prevents sperm binding to the zona pellucida and decreases female fertility.

## Conclusions

ZP2 is a major component of the zona pellucida to which sperm bind prior to penetrating the zona matrix and fusing with the egg. Following fertilization and cortical granule exocytosis, ZP2 is cleaved by ovastacin after which sperm do not bind to the zona pellucida. *Zp2*^*Mut*^ and *Astl*^*Null*^ mice documented that sperm binding to the surface of the zona pellucida is dependent on the cleavage status of ZP2, independent of fertilization and cortical granule exocytosis [27, 34]. In each of these mutant lines, ZP2 is uncleaved in the zona pellucida surrounding ovulated eggs which can be fertilized *in vitro* and *in vivo*. After fertilization and cortical granule exocytosis, ZP2 remains uncleaved and sperm can bind *de novo* to the zona matrix surrounding zygotes derived from *Zp2*^*Mut*^ and *Astl*^*Null*^ female mice. Although at risk, the additional post-fertilization block to sperm fusing with the egg plasma membrane [1, 2] and the relatively few sperm (<10 sperm/egg) that reach the egg in the ampulla of the oviduct [39, 40] help protect *Zp2*^*Mut*^ and *Astl*^*Null*^ females from polyspermy.

The observed partial cleavage of ZP2 in the *Astl*^*Δ/Δ*^ is puzzling and could reflect a continuous release of low concentrations of constitutively secreted ovastacin during oocyte growth rather than the abrupt release of higher concentrations of the enzyme thought to accompany cortical granule exocytosis at fertilization. This formulation is consistent with earlier observations in which incubation of ovulated eggs in the absence of serum proteins caused a ‘hardening’ reaction that prevented sperm binding to the zona pellucida and fertilization [41]. The molecular basis of this phenomenon has recently been ascribed to fetuin-B, a member of the cystatin superfamily of protease inhibitors that are secreted by the liver and circulate as serum proteins. Fetuin was reported to inhibit premature cleavage of ZP2 [38] and this activity has been attributed more precisely to fetuin-B which is present at low levels in follicular fluid [42]. It has been proposed that a minimal amount of fetuin-B is sufficient to inhibit the small amount of secreted ovastacin that escapes sequestration in cortical granules and prevent premature cleavage of ZP2. However, the ovastacin released from cortical granule exocytosis would overwhelm this fragile defense and cleave ZP2 to prevent post-fertilization sperm binding [43]. Similar to *Astl*^*Δ/Δ*^ mice, ZP2 is only partially cleaved in the zona matrix surrounding ovulated eggs from *Fetub*^*Null*^ mice and yet female mice are sub-fertile.

Thus, the timing of ZP2 cleavage by ovastacin is a critical determinant of whether or not sperm bind and fertilize eggs encased in the surrounding zona pellucida. Premature ZP2 cleavage (*Astl*^*Δ/Δ*^ or *Fetub*^*Null*^) prevents sperm binding and fertility, whereas delayed or absent ZP2 cleavage runs the risk of post-fertilization polyspermy. Eggs from *Astl*^*Null*^ females do not accumulate ovastacin in their cortical granules and are unable to cleave ZP2 following fertilization and cortical granule exocytosis. The ability of *Astl*^*mCherry*^ to rescue this phenotype and restore fertility confirms the role of ovastacin in the post-fertilization cleavage of ZP2 and provides a marker for future investigations into the biology of cortical granules. The cortical granule localization motif defined *in vitro* and confirmed *in vivo* by deletion of a 7 amino acids in the endogenous gene locus is highly conserved among mammals. Identification of binding partners to ovastacin should provide further insight into the molecular basis for the translocation of ovastacin to these unique subcellular organelles of female germ cells.

## Materials and Methods

### Establishment of *Astl*^*mCherry*^ transgenic mice

All mice were handled in compliance with the guidelines of the Animal Care and Use Committee of the National Institutes of Health under the Division of Intramural Research, National Institute of Diabetes and Digestive and Kidney Diseases approved animal study protocols. A summary of the transgenic mice used in these investigations is provided in S2 Table.

To construct the transgene, bacterial artificial chromosome (BAC) DNA (Life Technologies) that included mouse *Astl* (BMQ56H22) was transformed into SW102 bacterial cells containing the λ prophage recombineering system [44]. A PCR fragment (1331 bp) containing the galK operon flanked by 50 bp homologous to the *Astl* gene that would insert the galK gene at the stop codon in the 3’ region of the gene was amplified using Long Amp Taq Polymerase (New England Biolabs, Ipswich, MA). After digestion with DpnI and overnight gel purification (0.7% agarose, 15 V, 16 hr), the PCR fragment was electroporated into SW102 cells containing the BAC, and recombinants were selected by growth on minimal media with galactose. Using a clone from this first step, the galK cassette was replaced by recombineering with a second PCR fragment (880 bp) encoding mCherry with 50 bp homology arms. Clones were selected on minimal media with 2-deoxy-galactose and confirmed by DNA sequence. Finally, a NotI fragment containing the *Astl*^*mCherry*^ transgene was retrieved from the BAC with pl253, and the fidelity of coding regions was confirmed by DNA sequence.

After gel purification, the *Astl*^*mCherry*^ transgene was injected into the male pronucleus of >200 one-cell zygotes which were transferred to pseudopregnant female mice. Offspring were genotyped (S1b Fig) using tail DNA and *Astl* exon 9 oligonucleotides (S3 Table) that distinguished between the normal allele (268 bp) and the *Astl*^*mCherry*^ transgene (967 bp). From 30 pups, 6 founders were identified that passed the transgene through the germline. Three lines were maintained and the analysis of FVB/N-Tg(Astl-mCherry)1Dean is described herein. Tissue-specific expression of *Astl*^*mCherry*^ was determined by RT-PCR using total RNA from various tissues to make cDNA with SuperScript^®^ III First-Strand Synthesis System (Life Technologies). PCR analysis of cDNA was performed using primers (S3 Table) designed to span an exon-intron boundary.

### Establishment of *Astl*^*Δ/Δ*^ mice by CRISPR/Cas9

pMLM3613 (Addgene, Cambridge, MA #42251) expressing Cas9 was linearized by PmeI (New England Biolabs), purified with a PCR clean-up kit (Clontech Laboratories, Mountain View, CA) and *in vitro* transcribed with mMESSAGE mMACHINE T7 ULTRA (Life Technologies-Ambion, Carlsbad, CA). Double-stranded synthetic DNA targeting exon 2 of *Astl* (5’-GGACATCCCCGCAATTAACCAAGG-3’) was cloned into the pair of BsaI sites of pDR274 (Addgene, #42250) expressing sgRNA. After linearization by digestion with DraI, the plasmid was purified with the PCR clean-up kit and *in vitro* transcribed using MEGAshortscript T7 (Life Technologies-Ambion). After transcription, the Cas9 cRNA and the sgRNA were purified with MEGAclear kit (Life Technologies-Ambion) according to the manufacturer’s instruction and eluted in RNase-free water.

For zygote injection, B6D2_F1_ (C57BL/6 x DBA2) female mice were hormonally stimulated to ovulate (see below) and mated with B6D2_F1_ males. One-cell embryos were collected and injected with Cas9 cRNA (50 ng/μl), sgRNA (20 ng/μl) and donor oligo (20 ng/μl). The injected embryos were cultured in KSOM (Zenith Biotech, Guilford, CT) until the blastocyst stage and transferred into pseudopregnant CD1 female mice. The sequence of injected oligonucleotide was: 5’-TCTGGAGTCTGCAGTACCAGTGTTCCAGAAGGCTTCACTCCTGAGGGAAGCCCGGTATTTCAGAACCAAGGTGAGAACACGGGGCCACACTCCAAAGCCATGCTGAATGTGGACATGCGGAAAAGA-3’. The genotype of the *Astl*^*Δ*^ allele was initially determined by DNA sequence of tail DNA and subsequently by PCR using an oligonucleotide primer that bridged the deleted sequence (S3 Table).

### Oocyte, egg and embryo collection and culture

GV-intact oocytes from 4–6-wk-old female mice were collected by puncturing ovarian follicles in M2 medium (Sigma-Aldrich, St. Louis, MO) at 48 hr post-injection of 5 IU of equine gonadotropin hormone (eCG). Ovulated eggs and embryos from 4–6-wk-old female mice were collected before and after mating, respectively, in M2 medium after injection of 5 IU of eCG followed by 5 IU of human chorionic gonadotropin (hCG) 46–48 hr later. Embryos were subsequently cultured in KSOM (Zenith Biotech) at 37°C in 5% CO_2_ to obtain 1- and 2-cell embryos. To inhibit actin polymerization, the medium was supplemented with 100 μM CK-666 (Sigma-Aldrich), a cell permeable inhibitor of arp2/3. For individual experiments, 20–30 cells were used from 3 different animals and representative images were included in figures.

### Antibodies

A rabbit polyclonal antibody that binds a C-terminal peptide of ovastacin^395-408^ and monoclonal antibody M2c.2 that binds to the C-terminal region of ZP2 have been characterized previously [32][45]. The following antibodies and lectins were obtained commercially: LCA-FITC (Sigma-Aldrich); antibodies to GP73, calregulin and EEA1, Alex Fluor 488 goat anti-rabbit IgG (H+L)(Life Technologies-Invitrogen, Carlsbad, CA); Alexa Fluor 555 donkey anti-rabbit IgG (H+L) (Life Technologies-Invitrogen); DyLight 649 goat anti–rabbit IgG (H+L) (Life Technologies-Invitrogen); and goat anti-rat IgG-HRP (Santa Cruz).

### Plasmid construction, cRNA *in vitro* transcription and microinjection

pCS2+/UtrCH-EGFP (Addgene, #26737) was linearized with NsiI and *in vitro* transcribed using SP6 mMESSAGE mMACHINE (Life Technologies-Ambion, AM1340). cRNA was purified by MEGAclear (Life Technologies-Ambion). Full-length and truncated ovastacin open reading frames were inserted into the pmCherry-N1 vector (Clontech Laboratories). Capped cRNAs were synthesized from PCR templates using T7 mMessage mMachine (Life Technologies-Ambion), and purified with MEGAclear (Life Technologies-Ambion). Microinjection was performed in M2 medium (Zenith Biotech) using a TransferMan NK2 micromanipulator (Eppendorf, Hauppauge, NY). Typically, 10–12 pl (4% of the oocyte volume) of 0.5–1.0 μg/μl cRNA was injected into oocytes. For each cRNA construct, 20–30 GV-intact oocytes from 3 mice were injected, incubated in M2 media (37°C, 5% CO_2_) for 6 hr prior to fixation and imaging by confocal microcopy. Variations in the intensity of the mCherry signal were noted among oocytes injected with the same construct and those with the strongest signals were selected for fixation and imaging by confocal microscopy using similar settings.

### Immunofluorescence and confocal microscopy

GV-intact oocytes, ovulated eggs or embryos (20–30) were fixed in 4% paraformaldehyde (PFA) for 30 min, permeabilized in 0.5% Triton X-100 for 20 min, and blocked in SuperBlock (Piercenet, Thermo-Fisher Scientific, Rockford, IL) for 1 hr at room temperature. Samples were incubated with primary antibody (1:100) for 1 hr at room temperature or 4°C overnight. After three washes in 0.3% PVP/PBS containing 0.1% Tween 20 and 0.01% Triton X-100 for 5 min each, oocytes or embryos were incubated with secondary antibody (1:200) for 1 hr at room temperature. For LCA staining, eggs or embryos were stained with LCA-FITC (1:100) for 1 hr at room temperature. After three washes in 0.3% PVP/PBS containing 0.1% Tween 20 and 0.01% Triton X-100 for 5 min each, samples were mounted in PBS containing Hoechst 33342 (1μg/ml). Confocal laser-scanning images were obtained using similar settings within experiments on an LSM 510 confocal microscope (Carl Zeiss AG, Jena, Germany), with a 63 x 1/2 W objective and exported as full-resolution TIF files and processed in Photoshop (Adobe Systems, San Jose, CA) to adjust brightness and contrast.

### Time-lapse imaging

Time-lapse imaging was obtained with the LSM 510 confocal microscope equipped with a Plan Apochromat 40×, 1.2 NA water immersion objective. mCherry was excited with a 561-nm laser line and detected with a 575–615-nm band pass. Ovulated eggs (10–20 from 3 animals) were collected, followed by removal of zonae pellucidae (see below), and placed in HTF medium supplemented with 5 ng/ml Hoechst 33342 (Life Technologies-Molecular Probes, Eugene, OR) on a gridded cover glass bottom dish (MatTek, Ashland, MA Cat. No. P35G-1.5-7-C-grid). The dish was placed in a humidified chamber (5% CO_2_, 37°C) attached to the microscope and inseminated with 1 x 10^4^ ml^-1^ capacitated sperm.

### Immunoblot analysis

GV-intact oocytes, ovulated eggs and two-cell embryos (10–15) were lysed in 4x LDS (lithium dodecyl sulfate) sample buffer with 10x reducing reagent (Life Technologies-Invitrogen), separated on 12% Bis-Tris precast gels, transferred to nitrocellulose membranes (Life Technologies-Invitrogen), blocked in 5% nonfat milk in TBS (Tris buffered saline, pH 7.4) with 0.1% Tween 20 (TBST) for 1 hr at room temperature, and then probed with 1:500–1:1,000 dilution of primary antibodies at 4°C overnight. On the following day, blots were incubated with a 1:10,000 dilution of secondary antibodies conjugated to HRP (horse radish peroxidase). Chemiluminescence was performed with ECL Plus (Piercenet) and signals were acquired by a Luminescent Image Analyzer LAS-3000 (Fujifilm, Valhalla, NY) or with BioMax XAR film (Kodak, Rochester, NY).

### Sperm binding assay

Caudal epididymal sperm were isolated from wild-type ICR mice and placed under oil (EMD Millipore, Billerica, MA) in human tubal fluid (HTF) medium (Zenith Biotech) previously equilibrated with 90% N_2_, 5% O_2_, 5% CO_2_ and capacitated by an additional 1 hr of incubation at 37°C. Sperm binding to ovulated eggs or two-cell embryos isolated from wild-type, *Astl*^*Null*^, *Astl*^*Rescue*^, *Astl*^*+/Δ*^ and *Astl*^*Δ/Δ*^ mice was observed using capacitated sperm and wild-type 2-cell embryos as a negative wash control. Samples were fixed in 4% PFA for 30 min, stained with Hoechst 33342. Bound sperm were quantified from z projections acquired by confocal microscopy [46], and results reflect the mean ± s.e.m. from at least three independently obtained samples, each containing 6–12 mouse eggs/embryos. Statistical differences were determined by the 2-tailed Student’s T-test.

### *In vitro* fertilization

The zona pellucida of eggs was removed after 5 min incubation in 100 μl of acid Tyrode's solution (Sigma) and then washed 3 times in fresh M2 medium. Cauda epididymides were lanced in a dish of HTF to release sperm that were capacitated for 1 hr (37°C with 90% N_2_, 5% O_2_, 5% CO_2_) and added to zona-intact or zona-free eggs (30 from 3 different animals) at a concentration of 4 x 10^5^ ml^-1^ sperm in 100 μl HTF for 5 hr at 37°C, 5% CO_2_. The presence of two pronuclei was scored as successful fertilization. Statistical differences were determined by the 2-tailed Student’s T-test.

## Supporting Information

S1 Fig *Astl*^*mCherry*^ transgenic mice. **(a)** Annotated representation of the *Astl*^*mCherry*^ transgene (upper) and the *Astl*^*Null*^ allele (lower) with the endogenous *Astl* allele. **(b)** PCR genotyping of tail DNA isolated from wild-type (Ctrl), *Astl*^*Null*^ (Null), *Astl*^*mCherry*^, and *Astl*^*mCherry*^; *Astl*^*Null*^ (Rescue) mice using primer pairs (1), (2) and (3) in **(a)**. Molecular mass (kB) on left. **(c)** Total RNA was extracted from brain (Br), heart (H), kidney (K), liver (Li), lung (Lu), spleen (S), uterus (U), ovary (O) and testis (T), and analyzed by RT-PCR with primers (S3 Table) to detect normal and *Astl*^*mCherry*^ transcripts. GAPDH was used to as a load control and to ensure integrity of RNA. C, water control. Molecular mass (kB) on left.
(TIF)

S2 Fig Localization of ovastacin in growing oocytes, ovulated eggs and zygotes from *Astl*^*mcherry*^ mice. **(a)** Growing *Astl*^*mCherry*^ oocytes (50–70 μm) were fixed and stained with antibodies specific to the endoplasmic reticulum (GP73), the Golgi apparatus (calregulin) and endosomes (EEA1) prior to imaging by confocal and DIC microscopy. Arrows, co-localization of marker and ovastacin. Scale bar, 20 μm. **(b)** Reversal of cortical granule free domain (CGFD) by inhibition of actin nucleation and cap formation. Ovulated eggs from *Astl*^*mCherry*^ mice were incubated for 3 hr with (lower panels) or without (upper panels) CK666 to inhibit Arp2/3. Eggs were stained with Hoechst prior to confocal and DIC microscopy. Scale bar, 20 μm. **(c)** Time-lapse images of cortical granule exocytosis after insemination of zona-free *Astl*^*mCherry*^ eggs (0 min) with capacitated sperm until formation of pronuclei in 1C zygotes (300 min). Eggs/embryos were imaged by confocal and DIC microscopy at the designated times after fixation and staining with Hoechst. PB, polar body. Scale bar, 20 μm.
(TIF)

S3 Fig Deletion mutation of *Astl* using CRISPR/Cas9. **(a)** Schematic representation of Cas9 targeted with single-stranded guide RNA (ssRNA) to exon 2 of *Astl* 5’ of the PAM (protospacer adjacent motif) to cut the double-stranded DNA with the RuvC and HNH sites. **(b)** Schematic representation of double-stranded donor DNA (126 bp) with a 21 bp (encodes ovastacin^52-58^) deletion used for homology directed DNA repair of the Cas9 induced DNA cleavage in exon 2 of *Astl*. **(c)** Genotype of tail DNA from 7 pups (AS1-7) derived from 1C zygotes injected with single-stranded guide RNA (20 ng/μl), RNA encoding Cas9 (50 ng/μl) and HDR oligonucleotide (20 ng/μl). The lower band in AS3 (**d**, left) and AS6 (**e**, left) were sequenced to confirm the 21 bp deletion. The middle band contained a single cytosine deletion in AS3 (**d**, right) and a single adenosine insertion in AS6 (**e**, right). The upper bands in AS3 and AS6 represent a heteroduplex of the two alleles migrating at a slower mobility. A c/t polymorphism is present in intron 2 of *Astl*.
(TIF)

S1 Table Fertility of *Astl* mutant female mice. (DOCX)

S2 Table Mouse alleles and proteins. (DOCX)

S3 Table Primers. (DOCX)
