# Supplementary figures and images for: A Unique Egg Cortical Granule Localization Motif Is Required for Ovastacin Sequestration to Prevent Premature ZP2 Cleavage and Ensure Female Fertility in Mice
Source: PLoS Genet. 2017 Jan 23;13(1):e1006580. doi: 10.1371/journal.pgen.1006580 (PMC5293279; doi:10.1371/journal.pgen.1006580)

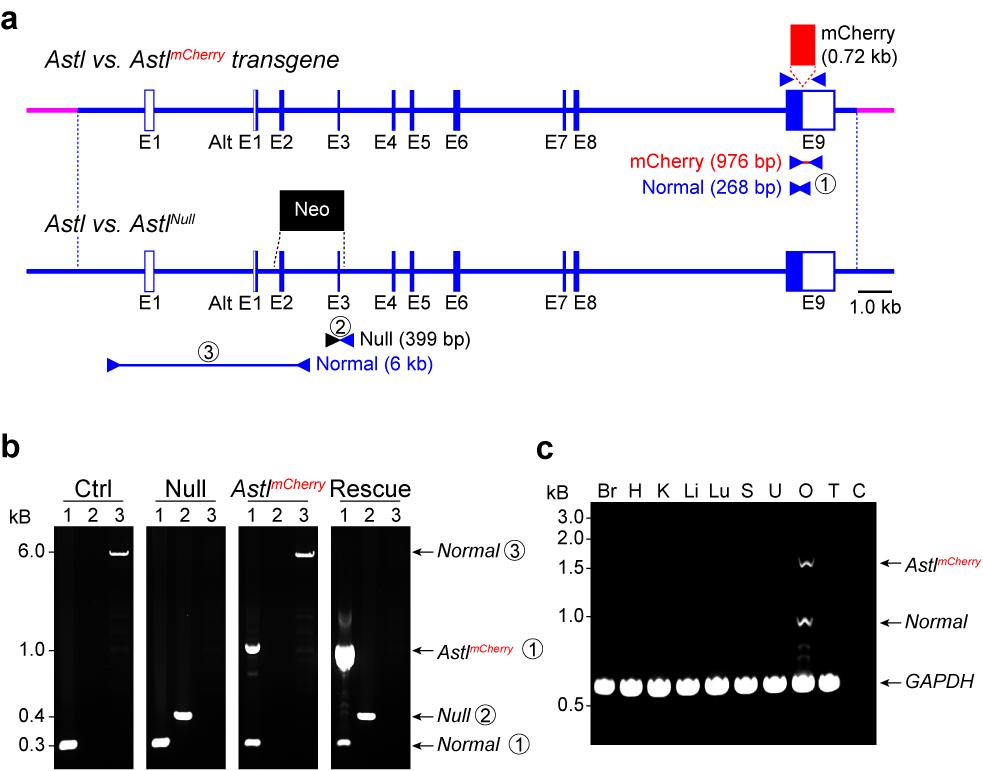

Supplement: S1 Fig — (a) Annotated representation of the AstlmCherry transgene (upper) and the AstlNull allele (lower) with the endogenous Astl allele. (b) PCR genotyping of tail DNA isolated from wild-type (Ctrl), AstlNull (Null), AstlmCherry, and AstlmCherry; AstlNull (Rescue) mice using primer pairs (1), (2) and (3) in (a). Molecular mass (kB) on left. (c) Total RNA was extracted from brain (Br), heart (H), kidney (K), liver (Li), lung (Lu), spleen (S), uterus (U), ovary (O) and testis (T), and analyzed by RT-PCR with primers (S3 Table) to detect normal and AstlmCherry transcripts. GAPDH was used to as a load control and to ensure integrity of RNA. C, water control. Molecular mass (kB) on left. (TIF) [file pgen.1006580.s001.tif]

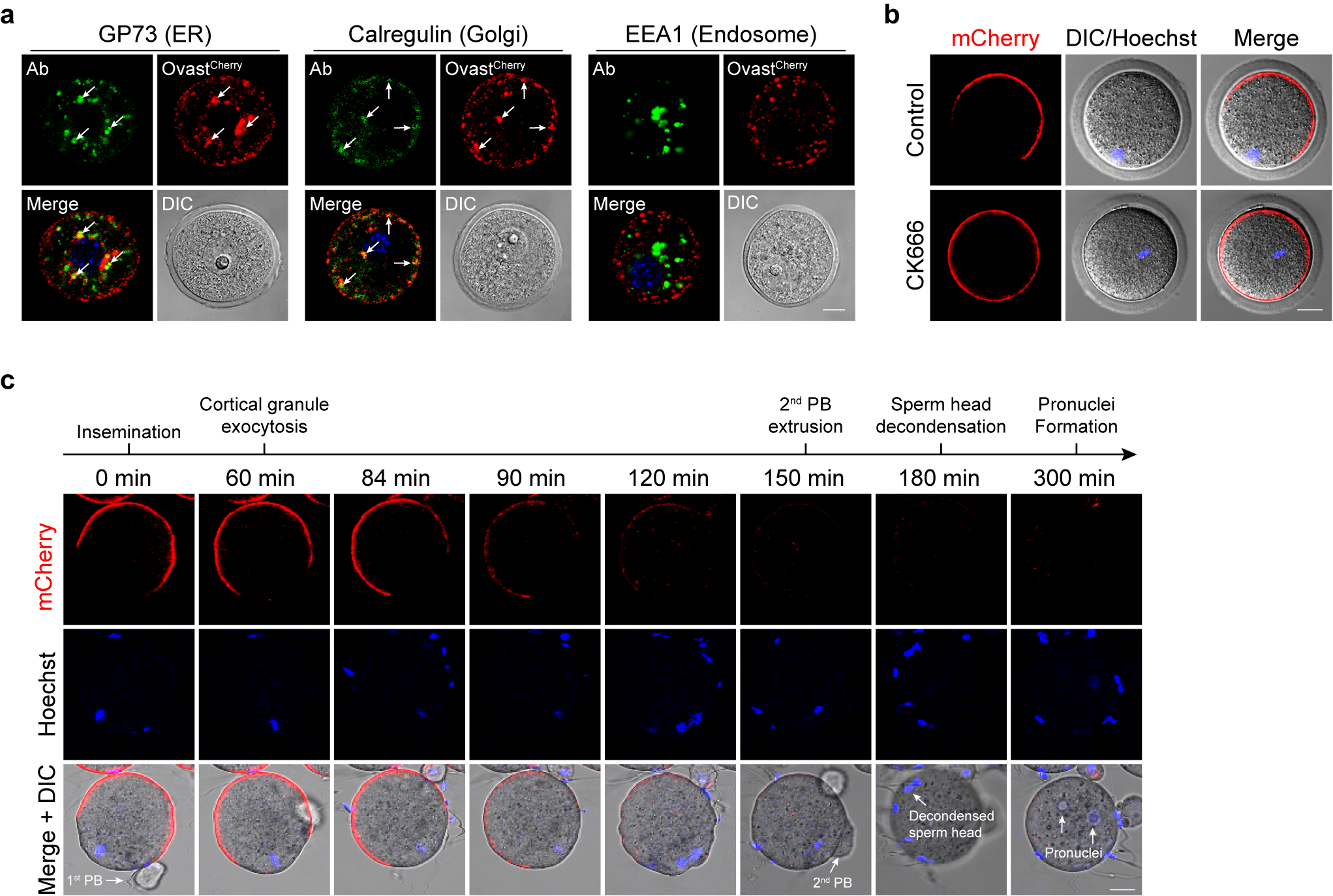

Supplement: S2 Fig — (a) Growing AstlmCherry oocytes (50–70 μm) were fixed and stained with antibodies specific to the endoplasmic reticulum (GP73), the Golgi apparatus (calregulin) and endosomes (EEA1) prior to imaging by confocal and DIC microscopy. Arrows, co-localization of marker and ovastacin. Scale bar, 20 μm. (b) Reversal of cortical granule free domain (CGFD) by inhibition of actin nucleation and cap formation. Ovulated eggs from AstlmCherry mice were incubated for 3 hr with (lower panels) or without (upper panels) CK666 to inhibit Arp2/3. Eggs were stained with Hoechst prior to confocal and DIC microscopy. Scale bar, 20 μm. (c) Time-lapse images of cortical granule exocytosis after insemination of zona-free AstlmCherry eggs (0 min) with capacitated sperm until formation of pronuclei in 1C zygotes (300 min). Eggs/embryos were imaged by confocal and DIC microscopy at the designated times after fixation and staining with Hoechst. PB, polar body. Scale bar, 20 μm. (TIF) [file pgen.1006580.s002.tif]

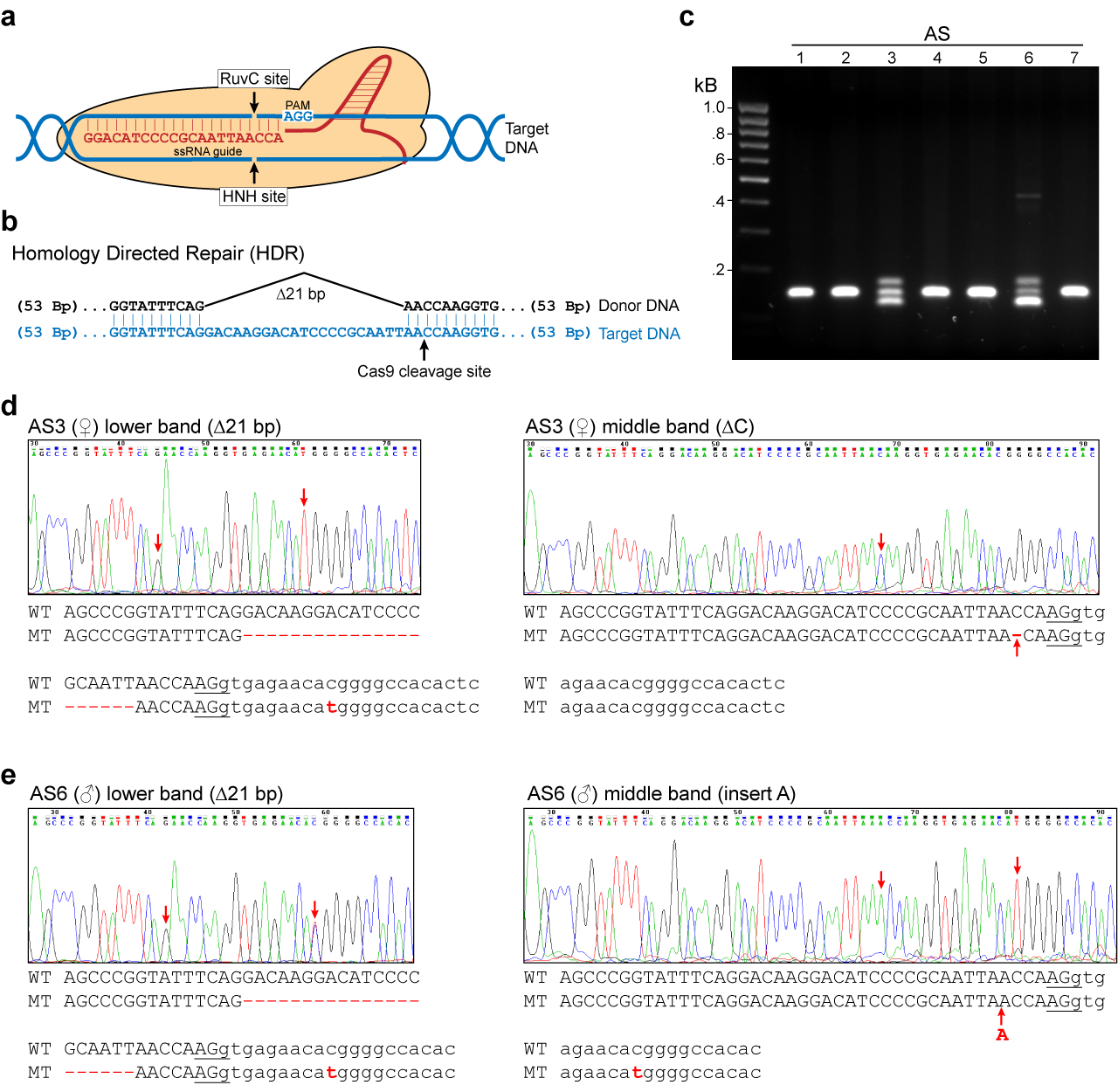

Supplement: S3 Fig — (a) Schematic representation of Cas9 targeted with single-stranded guide RNA (ssRNA) to exon 2 of Astl 5’ of the PAM (protospacer adjacent motif) to cut the double-stranded DNA with the RuvC and HNH sites. (b) Schematic representation of double-stranded donor DNA (126 bp) with a 21 bp (encodes ovastacin52-58) deletion used for homology directed DNA repair of the Cas9 induced DNA cleavage in exon 2 of Astl. (c) Genotype of tail DNA from 7 pups (AS1-7) derived from 1C zygotes injected with single-stranded guide RNA (20 ng/μl), RNA encoding Cas9 (50 ng/μl) and HDR oligonucleotide (20 ng/μl). The lower band in AS3 (d, left) and AS6 (e, left) were sequenced to confirm the 21 bp deletion. The middle band contained a single cytosine deletion in AS3 (d, right) and a single adenosine insertion in AS6 (e, right). The upper bands in AS3 and AS6 represent a heteroduplex of the two alleles migrating at a slower mobility. A c/t polymorphism is present in intron 2 of Astl. (TIF) [file pgen.1006580.s003.tif]
