# Supplementary material for: A Unique Egg Cortical Granule Localization Motif Is Required for Ovastacin Sequestration to Prevent Premature ZP2 Cleavage and Ensure Female Fertility in Mice
Source: PLoS Genet. 2017 Jan 23;13(1):e1006580. doi: 10.1371/journal.pgen.1006580 (PMC5293279; doi:10.1371/journal.pgen.1006580)
Supplement: S1 Table — (DOCX) [file pgen.1006580.s004.docx]

|  | Wild-type | *Astl^+/Δ^* | *Astl^Δ/Δ^* |
| --- | --- | --- | --- |
| Unfertilized Eggs | 2.0 ± 0.5^1^ | 13.6 ± 0.8 | 16.3 ± 0.5 |
| 2C Embryos | 13.3 ± 0.7^1^ | 4.0 ± 0.8 | 0.6 ± 0.2 |
| Fertilization Rate | 87.7 ± 2.3%^2^ | 22.5 ± 4.0% | 0.8 ± 0.8% |
| Pups | 9.2 ± 2.5^3^ | 2.5 ± 0.8 | 2^4^ |

^1^average ± s.e.m. of unfertilized eggs/embryos from 5 female mice 40 hr after mating (1:1) with wild-type male mice.

^2^number embryos divided by the total number of embryos and unfertilized eggs

^3^average ± s.e.m. of pups from co-caged groups of wild-type, *Astl*^+^*^/Δ^* and *Astl^Δ/Δ^* female mice mated with wild-type male mice for 6 months.

^4^1 female (of eight) gave birth to 2 pups after 6 months of mating.
