# Supplementary material for: A Unique Egg Cortical Granule Localization Motif Is Required for Ovastacin Sequestration to Prevent Premature ZP2 Cleavage and Ensure Female Fertility in Mice
Source: PLoS Genet. 2017 Jan 23;13(1):e1006580. doi: 10.1371/journal.pgen.1006580 (PMC5293279; doi:10.1371/journal.pgen.1006580)
Supplement: S2 Table — (DOCX) [file pgen.1006580.s005.docx]

| **Mouse Line** | ***Astl* Allele** | **Protein** |
| --- | --- | --- |
| Wild-type | *Astl^+^* | ovastacin |
| *Astl* null | *Astl^Null^* or *Astl^-^* | ----- |
| *Astl* deletion | *Astl^Δ^* or *Astl^Δ52-58^* | ovastacin^Δ52-58^ |
| *Astl^mCherry^* transgenic | *Astl^mCherry^* | ovastacin^mCherry^ |
