# Supplementary material for: A Unique Egg Cortical Granule Localization Motif Is Required for Ovastacin Sequestration to Prevent Premature ZP2 Cleavage and Ensure Female Fertility in Mice
Source: PLoS Genet. 2017 Jan 23;13(1):e1006580. doi: 10.1371/journal.pgen.1006580 (PMC5293279; doi:10.1371/journal.pgen.1006580)
Supplement: S3 Table — (DOCX) [file pgen.1006580.s006.docx]

**Supplemental figures and tables**

**Fig S1. *Astl^mCherry^* transgenic mice.** **(a)** Annotated representation of the *Astl^mCherry^* transgene (upper) and the *Astl^Null^* allele (lower) with the endogenous *Astl* allele. **(b)** PCR genotyping of tail DNA isolated from wild-type (Ctrl), *Astl^Null^* (Null), *Astl^mCherry^*, and *Astl^mCherry^*; *Astl^Null^* (Rescue) mice using primer pairs (1), (2) and (3) in **(a)**. Molecular mass (kB) on left. **(c)** Total RNA was extracted from brain (Br), heart (H), kidney (K), liver (Li), lung (Lu), spleen (S), uterus (U), ovary (O) and testis (T), and analyzed by RT-PCR with primers (Table S3) to detect normal and *Astl^mCherry^* transcripts. GAPDH was used to as a load control and to ensure integrity of RNA. C, water control. Molecular mass (kB) on left.

**Fig S2. Localization of ovastacin in growing oocytes, ovulated eggs and zygotes from *Astl^mcherry^* mice.** **(a)** Growing *Astl^mCherry^* oocytes (50-70 μm) were fixed and stained with antibodies specific to the endoplasmic reticulum (GP73), the Golgi apparatus (calregulin) and endosomes (EEA1) prior to imaging by confocal and DIC microscopy. Arrows, co-localization of marker and ovastacin. Scale bar, 20 μm. **(b)** Reversal of cortical granule free domain (CGFD) by inhibition of actin nucleation and cap formation. Ovulated eggs from *Astl^mCherry^* mice were incubated for 3 hr with (lower panels) or without (upper panels) CK666 to inhibit Arp2/3. Eggs were stained with Hoechst prior to confocal and DIC microscopy. Scale bar, 20 μm. **(c)** Time-lapse images of cortical granule exocytosis after insemination of zona-free *Astl^mCherry^* eggs (0 min) with capacitated sperm until formation of pronuclei in 1C zygotes (300 min). Eggs/embryos were imaged by confocal and DIC microscopy at the designated times after fixation and staining with Hoechst. PB, polar body. Scale bar, 20 μm.

**Fig S3. Deletion mutation of *Astl* using CRISPR/Cas9. (a)** Schematic representation of Cas9 targeted with single-stranded guide RNA (ssRNA) to exon 2 of *Astl* 5’ of the PAM (protospacer adjacent motif) to cut the double-stranded DNA with the RuvC and HNH sites*.* **(b)** Schematic representation of double-stranded donor DNA (126 bp) with a 21 bp (encodes ovastacin^52-58­^) deletion used for homology directed DNA repair of the Cas9 induced DNA cleavage in exon 2 of *Astl*. **(c)** Genotype of tail DNA from 7 pups (AS1-7) derived from 1C zygotes injected with single-stranded guide RNA (20 ng/μl), RNA encoding Cas9 (50 ng/μl) and HDR oligonucleotide (20 ng/μl). The lower band in AS3 (**d**, left) and AS6 (**e**, left) were sequenced to confirm the 21 bp deletion. The middle band contained a single cytosine deletion in AS3 (**d**, right) and a single adenosine insertion in AS6 (**e**, right). The upper bands in AS3 and AS6 represent a heteroduplex of the two alleles migrating at a slower mobility. A c/t polymorphism is present in intron 2 of *Astl*.

**Table S1**. **Fertility of *Astl* mutant female mice.**

^1^average ± s.e.m. of unfertilized eggs/embryos from 5 female mice 40 hr after mating (1:1) with wild-type male mice.

^2^number embryos divided by the total number of embryos and unfertilized eggs

^3^average ± s.e.m. of pups from co-caged groups of wild-type, *Astl*^+^*^/Δ^* and *Astl^Δ/Δ^* female mice mated with wild-type male mice for 6 months.

^4^1 female (of eight) gave birth to 2 pups after 6 months of mating.

**Table S2.** **Mouse alleles and proteins.**

**Table S3. Primers.**
